# Health and Human Wellbeing in China: Do Environmental Issues and Social Change Matter?

**DOI:** 10.3389/fpsyg.2022.860321

**Published:** 2022-05-17

**Authors:** Wenjuan Zhao, Miao Chang, Lei Yu, Muhammad Tayyab Sohail

**Affiliations:** ^1^School of Environment, Tsinghua University, Beijing, China; ^2^China Petroleum Planning & Engineering Institute, Beijing, China; ^3^School of Public Administration, Xiangtan University, Hunan, China

**Keywords:** environmental issues, health, human wellbeing, China, development

## Abstract

How to mitigate greenhouse gas emission and achieve human development remain major sustainability issues, particularly in China. Empirical research on the effects of climate warming and social change on human health and wellbeing is quite fragmented. This study examines the impact of environmental issues and social changes on health and human wellbeing using a time series data of China from 1991 to 2020. Findings show that environmental issues have a negative impact on health and human wellbeing in long run. While the internet is a form of social change that tends to improve health and human wellbeing in the long run. FDI exerts a positive effect on human health, but it does not improve wellbeing in the long run. In contrast, financial development does not improve human health but it has a significant positive impact on wellbeing in the long run. Our empirical insights have important implications for achieving human wellbeing through the pursuit of environmental sustainability and social change.

## Introduction

Global warming has become a serious environmental issue that directly and indirectly damages human health and wellbeing. The escalation in environmental pollution has significantly attracted the attention of environmentalists, policymakers, and researchers (Wang et al., 2017). Emerging environmental issues are worsening public health and environmental balance (Chen et al., 2017; Zhao et al., 2019). The World Health Organization estimates the cost of climate warming on human health and reports that 250 thousand people will die worldwide each year from 2030 to 2050 (World Health Organization [WHO], 2013). Moreover, according to a report published in the New England Journal of Medicine by Andrew Haines, the global death toll from rising temperatures is “much higher” than predicted by the World Health Organization (Haines and Kristie, 2019). The 2021 report of Countdown on health and climate change: code red for a healthy future released by the Lancet pointed out that nearly 350 thousand people around the world died of high temperature-related diseases in 2019 (Romanello et al., 2021). In the case of China, Mei et al. (2020) reported that economic growth has increased to a large extent during the last few years but the climate warming has also deteriorated on a large scale.

Existing literature reported that environment, lifestyle, heredity, and health care are major determinants that affect human health. With the increasingly serious environmental issues, the detrimental impacts of carbon emission on human health have been explored by many researchers in recent years (Matus et al., 2012; Sen and Mukherjee, 2014; Li and Zhou, 2020; Yuan et al., 2020; Lei et al., 2021). The literature provides a negative linkage between carbon emission and health outcomes (Farooq et al., 2019; Sarwar et al., 2019).

Studies have been done in exploring the transmission channels of the impacts of environmental issues on human health. According to the sustainable development goals of the United Nations, human wellbeing is directly and indirectly associated with environmental conditions. It is argued that good quality ecological conditions provide better opportunities for human wellbeing, hence producing positive associations between human wellbeing and good quality environmental conditions (Sohail et al., 2019a,Sohail et al., 2022b; Mahfooz et al., 2020; Yang et al., 2022). UNDP, and HDR, 2011 reported that major determinants of human development such as education, health, and wealth increase in the response of public demand for environmental resources due to urbanization, population density, globalization, and unsustainable pathways for production and consumption. Meanwhile, the central objective of sustainable development goals (SDGs) is to control intensifying pressure of environmental resources and to improve public wellbeing (Mahfooz et al., 2019; Sohail et al., 2021b; Olabi et al., 2022).

Two diverse approaches have been discussed in the literature related to environmental quality and human wellbeing. According to the first approach, societies can get win-win solutions that support public wellbeing and environmental quality. Given the second approach, the actions related to enhancing environmental quality adversely influence human wellbeing. Although environmental quality and public wellbeing have gained attention from stakeholders, environmentalists, and researchers, while transmission mechanism between environmental performance and human wellbeing has not been fully explored yet. Several studies have examined the association between economic development and environmental quality and relate human wellbeing with economic growth. However, various authors denoted that education and life expectancy which are major determinants of the human development index are highly correlated with mortality due to environmental issues related to calamities (Kampa and Castanas, 2008; Sohail et al., 2015; Sohail et al., 2019b; Sohail et al., 2021c; Ullah et al., 2021; Bai et al., 2022; Sohail et al., 2022a).

In the last few decades, life expectancy is improving throughout the world but many economies are still unable to attain a better quality of human health. To improve human health there is a need to promote such strategies that support the alleviation of health issues. Various researchers consider social change as a vital determinant to improve public health outcomes. Social change is mainly measured by information and communication technology and the use of the internet. Health care specialists use the internet to explore information regarding health care, for purpose of communication, and foreign collaborations (Ali et al., 2020; Sohail et al., 2021a). Chetley et al., 2006 study denoted that the internet is capable of improving healthcare and health outcomes in developing economies. Additionally, it can ease communication between healthcare experts and patients (Bankole et al., 2013; Yen et al., 2019; Sohail et al., 2021a). ICT helps in improving public utilities, efficiency, and management of governments. It is argued that the internet affects almost every field of human life. A vast body of literature has explored the impact of the internet on the GDP and productivity of economies (Dedrick et al., 2013; Sohail et al., 2020; Liu et al., 2022). But, the impact of the internet on human wellbeing has not been explored quite adequately (Sohail et al., 2014; Martin, 2016; Kim et al., 2021). The literature identified various determinants of human wellbeing such as access to ICT, community environment, social environment, lifestyle, demographic factors, and social inclusion. Neumayer (2012) denoted that the impact of the internet on human wellbeing can be explained through the channel of the capabilities approach. Human wellbeing is directly determined by the capability of individuals to pursue their objectives. Based on this approach, various studies reported that the use of the internet is beneficial for human health and human wellbeing (Chiao and Chiu, 2016; Sohail et al., 2022a). There exist several studies that empirically investigated the link between environmental pressures, social change, and health (Narayan et al., 2008; Correia et al., 2013; Meghisan and Toma, 2017; Sohail et al., 2019a). However, none of the studies has explored the impact of environmental issues and social change on health and human wellbeing in the case of China.

Different from the earlier studies, understanding the linkages between environmental issues, social change, human health, and human wellbeing is important for China where multiple social, environmental, and economic drivers are quite active in the depletion of human health and human wellbeing. In this light, our study aims to explore the impact of environmental performance and social change on human health and human wellbeing in China for the period 1991–2020. The study makes contributions in the following manners. The novel contribution is that the study explores the transmission channels between environmental issues, health, and human wellbeing. Existing studies focused on the relationship between environmental performance and health outcomes while considering the nexus between environmental performance and human wellbeing in the short and long term. Another contribution is that the study explores the impact of social change on human health and wellbeing in the short and long term. The study possesses both practical applications and theoretical meaning. The study will help governments and policymakers in designing policies to solve environmental problems actively and regulate ICT diffusion that leads to resource allocation and encourage economic growth that simultaneously enhances human wellbeing and human health.

## Materials and Methods

Environmental issues are adversely related to human health. The influence of air pollutants on health and human wellbeing has been a stimulating subject and gained much attention in theoretical and empirical research over the last 50 years. In accordance with the theory, Haines et al. (2006) and Castellacci and Tveito (2018) noted that climate change and social change have played a role in human health and wellbeing. To estimate the impact of greenhouse gas emission and the internet on human wellbeing, we estimate the following empirical models which are reliable to previous empirical studies such as Castellacci et al., 2017 and Kassouri and Altıntaş (2020). The models are written as:


(1) $${\text{Health}}_{\text{t}}=\delta_{0}+\delta_{1}\,{\text{EP}}_{\text{t}}+\delta_{2}\,{\text{Internet}}_{\text{t}}+\delta_{3}\,{\text{FDI}}_{\text{t}}+\delta_{4}\,{\text{FD}}_{\text{t}}+\varepsilon_{\text{t}}$$

(2) $${\text{HDI}}_{\text{t}}=\delta_{0}+\delta_{1}\,{\text{EP}}_{\text{t}}+\delta_{2}\,{\text{Internet}}_{\text{t}}+\delta_{3}\,{\text{FDI}}_{\text{t}}+\delta_{4}\,{\text{FD}}_{\text{t}}+\varepsilon_{\text{t}}$$

Where Health_t_ is population health and HDI_t_ is the human development index that depends on greenhouse gas emission (EP), internet users (Internet), inward foreign direct investment (GDP), and financial development (FD). Greenhouse gas emissions have direct adverse impacts on population health that also reduces human wellbeing, thus estimates of δ_1_ to be negative in Equations (1 and 2). Internet is an effective way to improve health and wellbeing, thus sign of δ_2_ is likely to be positive. The population health and wellbeing impacts caused by FDI and financial development can have favorable economic and social effects, thus δ_3_ and δ_4_ would be positive. Our estimates of δ_1_, δ_2_, δ_3_, and δ_4_ reflect long-run effects of exogenous variables on health and wellbeing in Equations (1 and 2), which is ignoring short-run effects. To assess long and short-run effects in one step, we re-write specifications (1 and 2) in an error-correction format as follows:


(3) $$\Delta \,{\mathrm{Health}}_{\text{t}}=\delta_{0}+\,\sum_{\text{k}=1}^{\text{n}}\beta_{1\,\text{k}}\,\Delta \,{\mathrm{Health}}_{\text{t}-\text{k}}+\,\sum_{\text{k}=0}^{\text{n}}\beta_{2\,\text{k}}\,\Delta \,{\mathrm{EP}}_{\text{t}-\text{k}}$$

$$\;+\sum_{\text{k}=1}^{\text{n}}\beta_{3\,\text{k}}\,\Delta \,{\mathrm{Internet}}_{t-\text{k}}+\,\sum_{\text{k}=0}^{\text{n}}\beta_{4\,\text{k}}\,\Delta \,{\mathrm{FDI}}_{2,\text{t}-\text{k}}$$

$$\;+\sum_{\text{k}=1}^{\text{n}}\beta_{5\,\text{k}}\,\Delta \,{\mathrm{FD}}_{t-\text{k}}+\delta_{1}\,{\text{Health}}_{\text{t}-1}+\delta_{2}\,{\text{EP}}_{\text{t}-1}$$

$$\;+\delta_{3}\,{\text{Internet}}_{\text{t}-1}+\delta_{4}\,{\text{FDI}}_{2,\text{t}-1}$$

$$\;+\delta_{5}\,{\text{FD}}_{\text{t}-1}+\lambda .{\mathrm{ECM}}_{\text{t}-1}+\varepsilon_{\text{t}}$$

(4) $$\Delta \,{\mathrm{HDI}}_{\text{t}}=\delta_{0}+\,\sum_{\text{k}=1}^{\text{n}}\beta_{1\,\text{k}}\,\Delta \,{\mathrm{HDI}}_{\text{t}-\text{k}}+\,\sum_{\text{k}=0}^{\text{n}}\beta_{2\,\text{k}}\,\Delta \,{\mathrm{EP}}_{\text{t}-\text{k}}$$

$$\;+\sum_{\text{k}=1}^{\text{n}}\beta_{3\,\text{k}}\,\Delta \,{\mathrm{Internet}}_{t-\text{k}}+\,\sum_{\text{k}=0}^{\text{n}}\beta_{4\,\text{k}}\,\Delta \,{\mathrm{FDI}}_{2,\text{t}-\text{k}}$$

$$\;+\sum_{\text{k}=1}^{\text{n}}\beta_{5\,\text{k}}\,\Delta \,{\mathrm{FD}}_{t-\text{k}}+\delta_{1}\,{\text{HDI}}_{\text{t}-1}+\delta_{2}\,{\text{EP}}_{\text{t}-1}$$

$$\;+\delta_{3}\,{\text{Internet}}_{\text{t}-1}+\delta_{4}\,{\text{FDI}}_{2,\text{t}-1}$$

$$\;+\delta_{5}\,{\text{FD}}_{\text{t}-1}+\lambda .{\mathrm{ECM}}_{\text{t}-1}+\varepsilon_{\text{t}}$$

A specification (3 and 4) resembles the famous ARDL model of the Pesaran et al. (2001). This model can fulfill our objective by providing short- and long-run estimates simultaneously without exerting any extra effort. Differentiating the short- and long-run estimates from specifications (3 and 4) is not a difficult task; the estimates attached to the first difference (Δ) variables provide the short-run results, and the long-run results can be picked from the estimates δ_2_−δ_5_ normalized on δ_1_. However, the long-run results are not considered genuine unless the test of co-integration i.e., ECM, supports them. Most of the panel co-integration methods work only if the variables included in the model are I(1). However, this method of estimation is quite advantageous because it does not require all the variables to be integrated in the same order. Therefore, the ARDL model can work well if the variables included in the model are a mixture of I(0) and I(1). The ARDL model can effectively control the issue of endogeneity because it also includes the short-run dynamic process. Lastly, the application of ARDL is not an issue in the case of a small sample size and provides efficient estimates. Lastly, robust and unbiased estimates of the model are confirmed by using different diagnostics and stability tests, such as lagrange multiplier (LM), Breusch-Pagan-Godfrey (BP), Ramsey RESET, and CUSUM and CUSUM-sq.

### Data

The study aims to explore the impact of environmental issues and social change on health and human wellbeing. From this perspective, the study uses time-series data for the period 1990–2020 for China. Table 1 reveals the information about symbols and definitions of variables and sources of data. Life expectancy at birth is used to measure health outcomes while human wellbeing is captured by the human development index. Environmental issues are measured by two proxy variables representing climate change, i.e., CO_2_ emissions and greenhouse gas emissions (including carbon dioxide emissions). CO_2_ emissions and greenhouse gas emissions data are taken into kilotons. The internet has been a tool for social change. Thus, we employed the internet as a proxy of social change. However, social change is measured by individuals using the internet as a percent of the total population. Besides these focused variables, the study has incorporated some control variables such as foreign direct investment and financial development index. The source of data for the human development index is UNDP and for the financial development index is IMF. The data for the remaining variables have been extracted from the World Bank.

**TABLE 1:** Definition’s and sources.

Variables	Definition’s	Sources
LE	Life expectancy at birth, total (years)	World Bank
HDI	Human development index	UNDP
CO_2_	CO_2_ emissions (kt)	World Bank
GEG	Total greenhouse gas emissions (kt of CO_2_ equivalent)	World Bank
Internet	Individuals using the Internet (% of population)	World Bank
FDI	Foreign direct investment, net inflows (% of GDP)	World Bank
FD	Financial development index	IMF

## Empirical Results

We are dealing with time-series data, it is mandatory to check the stationarity properties of data before performing regression analysis. In this regard, the study adopted PP and DF-GLS tests. Table 2 delivers the outcomes of both unit root tests. The findings of both DF-GLS test and PP test report that only FDI is stationary at level, while the rest of the variables are non-stationary at the level. However, these variables become stationary after taking their first difference. Based on the findings of unit root tests, the study has chosen the ARDL regression approach for further analysis. We have estimated four separate models. Table 3 demonstrates the short-run and long-run findings of ARDL models. Besides long-run and short-run findings, the study also reports findings of some important diagnostic tests which are required for confirming the stability of coefficient estimates of ARDL regressions.

**TABLE 2:** Unit root testing.

	PP		DF-GLS			
	I(0)	I(1)	Decision	I(0)	I(1)	Decision
LE	1.276	−2.654*	**I(1)**	–0.235	-1.654*	**I(1)**
HDI	0.225	−4.778***	**I(1)**	0.212	-4.125***	**I(1)**
CO_2_	–0.897	−2.854*	**I(1)**	–0.289	-2.788***	**I(1)**
GHE	–0.798	−2.654*	**I(1)**	–0.345	-2.574**	**I(1)**
Internet	2.255	−2.714*	**I(1)**	1.023	-1.742*	**I(1)**
FDI	−2.654*		**I(0)**	−1.724*		**I(0)**
FD	–0.512	−5.125***	**I(1)**	0.452	-5.385***	**I(1)**

***p < 0.01; **p < 0.05; *p < 0.1.

*Stationary at level i.e. I(0); stationary at first difference i.e. I(1).*

**TABLE 3:** ARDL estimates of health and human wellbeing.

	Health		Health		HDI		HDI	

	Coefficient	t-Stat	Coefficient	t-Stat	Coefficient	t-Stat	Coefficient	t-Stat
**Short-run**								
CO_2_	−0.603***	2.775			−0.034*	1.959		
CO_2_ (-1)	0.125	1.254			0.015	0.055		
CO_2_ (-2)					–0.005	1.414		
GHG			−1.069***	2.648			−0.060***	4.120
GHG (-1)			0.324	0.583			0.029*	1.721
GHG (-2)			0.051	0.139			–0.002	0.112
Internet	0.018*	1.944	0.019*	1.898	0.012***	3.516	0.013***	6.817
Internet (-1)	0.040**	2.224	0.042**	2.178	−0.004***	3.978	−0.002***	3.460
Internet (-2)	0.024**	2.278	0.025**	2.253	0.002****	3.391	0.001	0.091
FDI	0.055*	1.832	0.063*	1.763	0.015	0.134	0.011	0.580
FDI (-1)	0.003	0.193	0.003	0.229	–0.011	0.599	0.011**	2.154
FDI (-2)	0.048***	3.249	0.049**	2.949	0.001	0.526	–0.001	1.302
FD	0.214	0.411	0.103	0.181	0.004	0.096	0.007	0.256
FD (-1)	–0.698	1.502	–1.013	1.667	0.003	0.092	−0.044*	1.746
FD (-2)			0.364	0.661	0.045	1.251	–0.016	0.604
**Long-run**								
CO_2_	−1.420***	6.750			−0.086**	2.308		
GHG			−1.537***	5.542			−0.092**	2.146
Internet	0.018*	1.783	0.019*	1.888	0.009*	1.723	0.010*	1.840
FDI	0.739***	5.792	0.538***	4.763	0.010	1.291	0.011	1.466
FD	2.194	0.919	2.540	0.809	0.191*	1.892	0.170*	1.768
C	13.86*	1.766	20.91***	2.645	–0.572	1.321	–0.824	1.384
**Diagnostics**								
*F*-test	4.797*		14.25***		6.452***		6.542***	
ECM (-1)*	−0.308***	24.52	−0.315***	18.96	−0.302***	7.631	−0.289***	7.538
LM	0.412		0.452		1.015		1.025	
BP	1.485		1.523		0.456		0.654	
RESET	1.625		0.785		1.325		1.365	
CUSUM	S		S		S		S	
CUSUM-sq	S		S		S		S	

***p < 0.01; **p < 0.05; *p < 0.1.

Table 3 in model 1, long-run findings infer that the impact of CO_2_ emissions is significant and negative on health outcomes revealing that life expectancy tends to decline due to an increase in carbon emissions. It shows that a 1% intensification in CO_2_ emissions reduces life expectancy by 1.420% in the long run. Social capital reports a significant and positive impact on life expectancy in the long run confirming that the use of the internet contributes significantly to increasing health outcomes. It infers that a 1% increase in internet users results in improving life expectancy by 0.018% in the long run. The findings display that FDI also improves life expectancy in the long run as shown by the positive and significant coefficient estimate. In the short run, findings display a negative association between CO_2_ emissions and health and the positive impact of the internet and FDI on CO_2_ emissions.

In model 2, findings report the negative impact of greenhouse gas emissions on health outcomes in the long run. It is found that a 1% intensification in greenhouse gas emissions results in reducing life expectancy by 1.537% in the long run. It is found that the impact of the internet is positive and significant on life expectancy. It displays that a 1% upsurge in internet users increases life expectancy by 0.019% in the long run. FDI brings a significant and positive impact on life expectancy with a coefficient estimate of 0.538% in the long run. In the short run, findings display that greenhouse gas emissions bring significant and negative impacts on life expectancy, while the internet and FDI report a significant and positive impact on life expectancy.

In models 3 and 4, the impact of social capital and environmental performance has been explored on the human development index. The long-run findings in model 3 demonstrate that CO_2_ emissions report a negative impact on the human capital index as described by the negative coefficient estimate. It infers that an increase in CO_2_ emissions brings a significant reduction in human wellbeing. The findings display that 1% intensification in CO_2_ emissions causes 0.086% reduction in the human development index in the long run. The coefficient estimate for the internet is significant and positive confirming that an increase in social capital improves human wellbeing in the long run. It is observed that a 1% increase in internet users produces 0.009% increase in the human development index. The findings display that the financial development index produces significant improvement in life expectancy in the long run. In the short-run, it is found that CO_2_ emissions reduce human wellbeing while social capital enhances human wellbeing significantly. However, the impact of both control variables is insignificant on human wellbeing in the short run.

In model 4, long-run findings reveal that greenhouse gas emissions bring a significant reduction in human wellbeing. It is reported that 1% intensification in greenhouse gas emissions produces a 0.092% reduction in the human development index. The impact of social capital is significant and positive on the human development index in the long run. It reveals that a 1% increase on the internet increases human wellbeing by 0.010% in the long run. Financial development tends to increase human wellbeing in the long run as displayed by significant and positive coefficient estimates. In the short run, findings show that greenhouse gas emissions deteriorate human wellbeing as shown by the negative coefficient estimate of the human development index while the internet contributes significantly to improving human wellbeing as displayed by the positive coefficient estimate of the internet. The findings of diagnostics tests confirm the validity of the outcomes of our regression models. Long-run co-integration among variables is confirmed as shown by the results of F-stat and ECM findings. No issue of heteroskedasticity and autocorrelation is detected, and the stability condition is also fulfilled as described by the results of LM, BP, and CUSUM and CUSUM-sq tests. Moreover, the findings of the Ramsey RESET test confirm that the error terms are normally distributed.

## Discussion

Empirical findings show that greenhouse gas emissions have a negative impact on human health and wellbeing. This finding is also reliable with Auger et al. (2015), who noted that high temperatures due to climate change have a significant impact on infant mortality in Canada. Our finding is also backed by Tan et al. (2022), who suggest that greenhouse gas emissions have a negative impact on population health by reducing human wellbeing. This implies that a robust negative relationship existed between greenhouse gas emissions and health in China. This also means that greenhouse gas emissions have severe effects on economic and social performance, while the same finding is also reported by Sarwar et al. (2019). CO_2_ emissions enhance, directly and indirectly, health costs, which in turn reduces human health. Environmental issues are also reduced economic performance. Environmental pressures on the core element of human wellbeing, including education, health, and wealth via transmission channels of unstable pathways of production and consumption, urban expansion, and poverty. Theoretically, it is accepted that environmental issues influence human wellbeing directly as well indirectly through poverty and income inequality.

The study found that the internet improves health and human wellbeing in China. This finding is also backed by Castellacci et al., 2017, who noted that the internet improves human wellbeing via time-saving, economic activities, access to information, and social networking. This means that the internet increases the quality of life by generating new sources of income, this, in turn, improves human health. The internet can save energy consumption (Usman et al., 2021), and mitigate environmental greenhouse gas emissions, which can immediately affect human wellbeing. This infers that the internet fosters awareness of environmentally friendly activities, affecting the environment as well as human wellbeing (Ali et al., 2020). Thus, our finding indicates that the internet improves social and economic activities more efficiently in each digital economy. Internet affects human wellbeing by mediating a set of personal characteristics.

## Conclusion

Due to rapid growth, industrialization, and urbanization are increasing rapidly in China. Thus, the resultant climate change, the deterioration of public health, and the decline in ecological balance are becoming major restricting determinants of economic development that affect human wellbeing. Moreover, literature reports that the use of the internet influences public health. However, the potential effect of the internet on human wellbeing is still neglected. Several studies reported that environmental issues deteriorate human health and human wellbeing to a larger extent. Previous studies have explored the impact of environmental issues on human health or the impact of the internet of public health separately, while the extensive study on exploring the impact of climate change and internet use on human wellbeing in the context of developing economies is still very scarce. Keeping this in view, our study is examining the impact of climate change and social change on human health and human wellbeing for china over the period 1991–2020. The findings of the study display that greenhouse gas emissions and CO_2_ emissions negatively influence health outcomes in the long run, while the internet tends to enhance life expectancy in the long run. Additionally, findings reveal that greenhouse gas emissions and CO_2_ emissions exert a negative influence on human wellbeing while internet utilization significantly improves human wellbeing in the long run. The impact of CO_2_ emissions and greenhouse gas emissions is negative on human health and human wellbeing in the short run. The internet reports a significant and positive impact on human wellbeing and health in the short run. The findings of control variables display that foreign direct investment improves health outcomes in the long run. In contrast, financial development enhances human wellbeing in the long run.

## Implications and Limitations

Based on these findings, our study proposed several policy implications. This study has long-lasting theoretical implications for human health and wellbeing. Theoretically, the study has strengthened and enriches the importance of climate change, internet development, and social media. Practically, the study provides suggestions for the government of China to design such strategies that work for environmental sustainability, internet development, and human wellbeing. It is suggested that policymakers should adopt such policies that promote environmental sustainability and increase human development. There is a need to enhance eco-friendly technologies that help in increasing life expectancy. The government of China should enlarge the investment in research and development and should initiate health and environment-related research projects that bring green technological innovation. There is a need to promote financial development that helps in generating more investment and employment opportunities, thus improving human wellbeing. The government should focus on education, as an increase in education helps in improving the wellbeing of people. Government should focus on ensuring the sustainability of the environment, hence green technologies and renewable energy sources should be adopted for production and consumption purposes that generate relatively less environmental greenhouse gas and pollution. The government should more investments in the education sector to improve human wellbeing. These policy implications are not only for China but also can be adopted in other developing economies. It is suggested that human development and education can help in improving human health, thus governments should promote education.

The present study contains some limitations. The study is measuring health outcomes through life expectancy only. This research topic fascinates attention in this area for future research. In further studies, all other indicators of health should be considered. Physical wellbeing, mental wellbeing, and economic wellbeing are ignored and must be considered in future studies. The study is capturing environmental performance through greenhouse gas emissions and CO_2_ emissions, while other measures of environmental performance should be considered in future research. Future research may also extend the empirical analysis to panel data studies for developing and developed economies.

## Data Availability Statement

The raw data supporting the conclusions of this article will be made available by the authors, without undue reservation.

## Author Contributions

WZ: conceptualization, methodology, software, and writing—original draft. MC: supervision and final draft approval. LY: data collection and analyzing. MS: editing and data collection, visualization, and investigation. All authors contributed to the article and approved the submitted version.

## Conflict of Interest

The authors declare that the research was conducted in the absence of any commercial or financial relationships that could be construed as a potential conflict of interest.

## Publisher’s Note

All claims expressed in this article are solely those of the authors and do not necessarily represent those of their affiliated organizations, or those of the publisher, the editors and the reviewers. Any product that may be evaluated in this article, or claim that may be made by its manufacturer, is not guaranteed or endorsed by the publisher.
